# Receptor subunit compositions underly distinct potencies of a muscle relaxant in fast and slow muscle fibers

**DOI:** 10.3389/fphys.2022.1026646

**Published:** 2022-10-11

**Authors:** Manami Yamashita, Yoshihiro Egashira, Shuntaro Nakamura, Souhei Sakata, Fumihito Ono

**Affiliations:** Department of Physiology, Osaka Medical and Pharmaceutical University, Takatsuki, Japan

**Keywords:** acetylcholine receptor (AChR), muscle relaxant, neuromuscular junction, zebrafish, muscle type

## Abstract

A line of studies in the 1960s–1980s suggested that muscle relaxants do not work uniformly on all skeletal muscles, though its mechanism has not been clarified. We showed here that a classical non-depolarizing muscle relaxant pancuronium inhibits fast muscle fibers at lower concentration compared to slow muscle fibers in zebrafish. The difference of effective concentration was observed in locomotion caused by tactile stimulation as well as in synaptic currents of the neuromuscular junction induced by motor neuron excitation. We further showed that this difference arises from the different composition of acetylcholine receptors between slow and fast muscle fibers in the neuromuscular junction of zebrafish. It will be interesting to examine the difference of subunit composition and sensitivity to muscle relaxants in other species.

## Introduction

Skeletal muscle relaxants, first introduced to medical use in 1940s (Griffith and Johnson, 1942; Whitacre and Fisher, 1945), are now clinically indispensable for safe execution of surgeries (Murphy, 2018). They are also employed to alleviate pathological conditions, e.g., low back pain, neck pain, fibromyalgia, tension headache or myofascial pain syndrome (See and Ginzburg, 2008). A group of aminosteroids has been extensively developed and employed as muscle relaxants to block nicotinic acetylcholine receptors (AChRs) in the neuromuscular junctions (NMJs) (Shafer, 2011; Hunter, 2017), though some muscle relaxants work independent from AChRs. Muscle relaxants working on AChRs are often classified into two classes depending on the presence or absence of depolarizing activity. Non-depolarizing muscle relaxants, such as pancuronium, vecuronium and rocuronium, inhibit the nicotinic acetylcholine receptor at the neuromuscular junction by blocking the binding of acetylcholine. Onset of action is relatively slow, and clinical effects last longer compared to depolarizing agents (Das et al., 2022). Pancuronium was developed in the 1960s, inspired by an alkaloid from the plant, malouetine (Lewis et al., 1967). Vecuronium and rocuronium were subsequently developed, and are now more widely used because of their compatibility with the reversal reagent sugammadex (Naguib, 2007).

Skeletal muscle cells in vertebrates comprise basically two types, slow (red) and fast (white), with additional intermediate types or subtypes depending on the species and researchers (Scott et al., 2001; Ahmed and Ali, 2016). Fast muscle fibers use energy generated by glycolysis and produce strong contractions, though prone to fatigue. In contrast, slow muscle fibers contract weakly while their reliance on oxidative metabolism makes them resistant to fatigue.

Whether a particular muscle relaxant affects all skeletal muscles with identical potency and efficacy was examined in the skeletal muscles of cats, with an interest centered on its clinical implications (Bonta and Goorissen, 1968; Day et al., 1983). These studies reported muscle relaxants have different potencies among muscles comprising different proportions of fast/slow muscle fibers, e.g., between soleus and gastrocnemius, which led authors to propose that slow and fast muscle fibers may have different potencies for muscle relaxants. However, the underlying mechanism has never been proposed, arguably because these studies predated the molecular identification of AChRs (Noda et al., 1982). After the cloning of receptor subunit genes, which suggested that subunit compositions are uniform across all muscle fibers, the issue of the potentially different sensitivities between muscle fibers was never revisited.

AChRs in the neuromuscular junctions (NMJs) are heteropentamers composed of five muscle specific subunits: 2α1s, *β1*, *δ*, and *ε* (or *γ*). *α*1 and *β*1 henceforth will be designated as *α* and *β* respectively for simplicity. In most vertebrates, *γ* subunits are expressed early in development and are replaced by ε in mature stages (Mishina et al., 1986). Recent studies showed that AChRs in zebrafish skeletal muscles have two distinct sets of subunit composition. While AChRs in fast muscle fibers have classical composition of αβδε/γ, those in slow muscle fibers lack *ε/γ* subunits and are composed of *α*, *β* and *δ* subunits (Mongeon et al., 2011).

Given the difference in subunit compositions, we hypothesized that AChRs may underlie the different sensitivities of slow and fast muscle fibers. Unlike mammalian muscles in which slow and fast fibers are intermingled in a single muscle, they are spatially segregated in fish skeletal muscles (Devote et al., 1996), which offers a unique advantage to test this hypothesis. We examined the locomotion and synaptic physiology in zebrafish. In addition, we studied heterologously expressed AChR subunits, and showed that the distinct potencies of pancuronium in fast and slow muscles fibers are caused by the subunit composition difference.

## Materials and methods

### Fish lines and maintenance

Zebrafish were maintained in a self-circulating system at the Osaka Medical and Pharmaceutical University. All methods were carried out in accordance with relevant guidelines and regulations. All experimental protocols were approved by the IACUC at Osaka Medical and Pharmaceutical University.

### High-speed video recordings and kinematic analysis

The movements of embryos were recorded using a digital high-speed video camera (Kron Technologies) attached to a dissecting microscope (Olympus). All recordings were performed at 23°C–27°C. The intact embryo at 24 hpf was attached to a suction pipette and oriented for recording. As little suction as possible was applied to minimize tissue damage. Under this condition, spontaneous coiling was recorded at 100 frames/s. Touch response of 72 hpf larvae was recorded at 1,000 frames/s. A puff stimulus with a glass pipette was used to elicit escape behaviors. The puff pipette was positioned close to the tip of the fish tail, and positive pressure (30 psi, 20 ms) was applied using a pulse pressure device (Parker Hannifin). By manually measuring the head (or tail) turn angle (*θ*) for each frame, the head turn speed and distance traveled were calculated. 0–10 mM of pancuronium was dissolved in solution containing 0.006% sea salt and 0.01% methylene blue and used for experiments.

### Establishment of a transgenic zebrafish line Tg (hb9:tTAad, TRE:ChRFR-TagRFP)

The zebrafish hb9 promoter was used to drive the expression of the advanced tetracycline transactivator (tTAad), specifically in motor neurons (Egashira et al., 2022). The tTA response element (TRE) composite promoter that drives the expression of channelrhodopsin fast receiver (ChRFR) (Wang et al., 2009) fused with TagRFP was also cloned into the same plasmid. The Tet inducible system strongly enhanced the expression of ChRFR in Tg motor neurons, allowing reliable recording of EPCs by optogenetic stimulation. Tg fish were generated by co-injection of the purified plasmid (25 ng/μl) and tol2 transposase mRNA (25 ng/μl) into one-cell stage embryos as described previously (Kawakami et al., 2004).

### Zebrafish electrophysiology

Recordings of EPC in larval zebrafish was performed as previously described (Egashira et al., 2022). Whole-cell voltage clamp recording at a holding potential of −80 mV was performed from fast and slow muscles at room temperature (23°C–27°C). Tg (hb9:tTAad, TRE:ChRFR-TagRFP) zebrafish larvae (4–5 dpf) were anesthetized with 0.02% tricaine in standard extracellular solution containing 112 mM NaCl, 2.0 mM KCl, 10 mM HEPES, 10 mM glucose, 2 mM CaCl_2_, and 1 mM MgCl_2_ (pH 7.3–7.4). The skin, head, and internal organs were then removed. To minimize muscle contraction in response to optogenetic stimulation, the fish preparation was treated with 1 μM μ-conotoxin GIIIA (Alomone labs) for 5 min and then transferred to a recording chamber perfused with high Mg^2+^ extracellular solution, in which MgCl_2_ was 5 mM. To activate ChRFR, brief flash of light from a 470 nm high-power LED (LED Engine) was delivered through the 40× objective. Flash duration was controlled by Patchmaster software (HEKA). The patch pipette internal solution contained 120 mM K-methanesulfonate, 5 mM KCl, 5 mM EGTA, and 10 mM HEPES (pH 7.2–7.3). Data acquired with an EPC10 amplifier (HEKA) were digitized at 50 kHz and low-pass filtered at 3 kHz. The series resistance was typically ∼10 MΩ and was not compensated. Recordings with series resistance >15 MΩ or >25% change in series resistance were excluded from the analysis.

### *In vitro* synthesis of nicotinic acetylcholine receptor mRNA

cDNAs for the muscle subunits *α*1, *β*1, *δ*, *ε* were cloned from zebrafish (Mongeon et al., 2011). cDNA of each subunit was cloned into the pTNT vector (Promega) for *in vitro* transcription. The purified DNA plasmid was linearized at the BamHI site and *in vitro* transcribed with T7 RNA polymerase (mMESSAGE mMACHINE T7 Transcription kit; Thermo Fisher Scientific).

### Oocyte electrophysiology

Electrophysiology was performed with a two-electrode voltage clamp (TEVC) using OC-725C (Warner Instruments) as reported previously (Hashimoto et al., 2022). ND 96 solution consisting of (in mM) 5 HEPES, 96 NaCl, 2 KCl, 1.8 CaCl_2_, 1 MgCl_2_, pH7.5 was used as the bath solution.

### Statistics

All data were expressed as mean ± SEM. Student’s *t*-test, paired *t*-test, Student’s *t*-test was used to determine the significance of difference. For multiple comparison test, *p*-value was corrected with Bonferroni.

## Results

### Effects of pancuronium on zebrafish locomotion

In zebrafish embryos at 24 h post fertilization (hpf), tactile stimulation induces coiling, which consists of 1-3 alternating contractions of the trunk. This locomotion depends on slow muscles, because mutants lacking functional fast muscles display normal coiling (Naganawa and Hirata, 2011; Zempo et al., 2020). In contrast, burst swimming after 48 hpf depends on fast muscles (Naganawa and Hirata, 2011).

**FIGURE 1:**
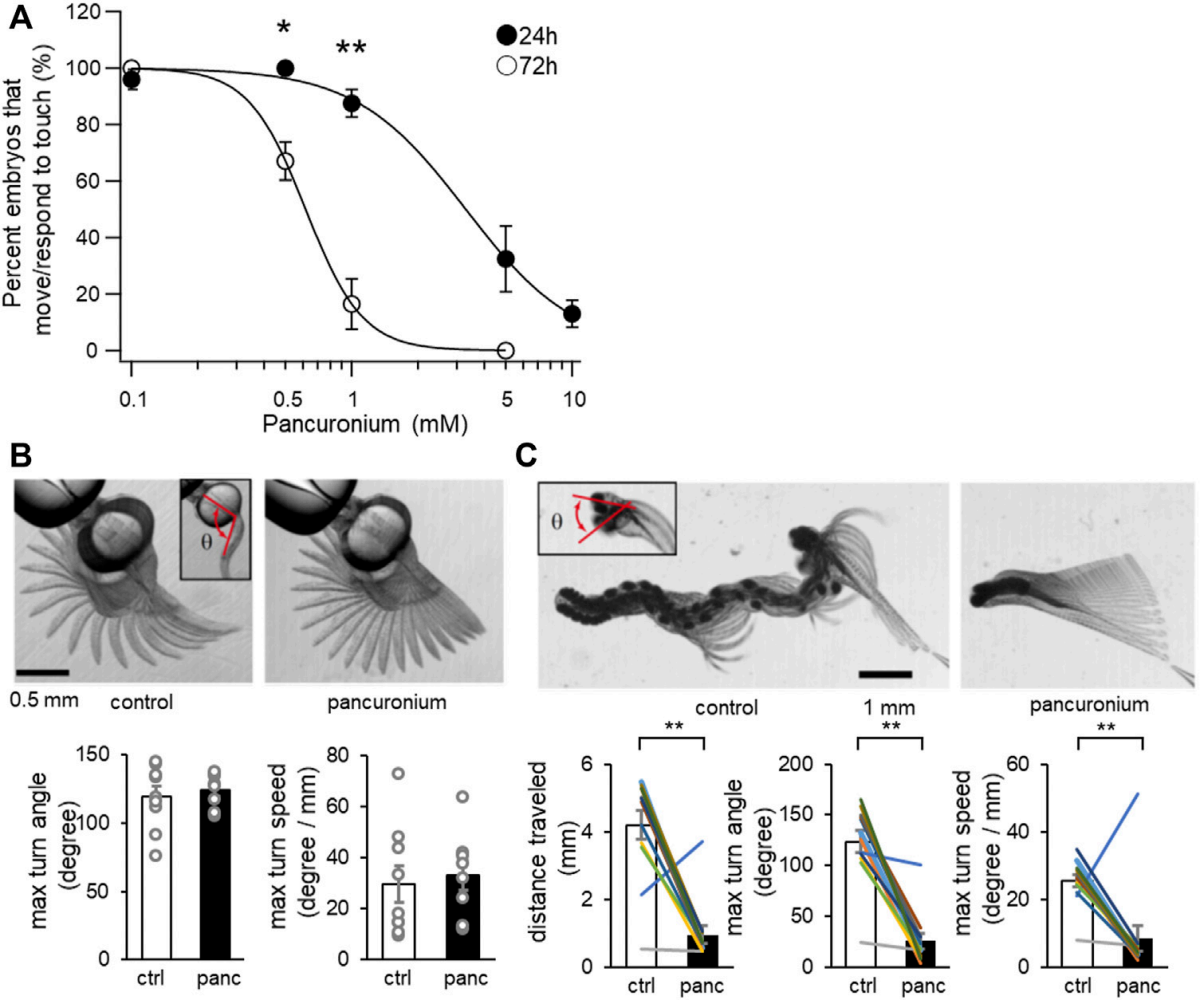
The effect of pancuronium on locomotor activity of zebrafish larvae. **(A)** Concentration dependent effects of pancuronium on locomotor activity of zebrafish larvae at 24 and 72 hpf. (*p < 0.05, **p < 0.01, Student’s *t*-test with bonferroni correction). **(B)** Superimposed images of spontaneous coiling at 24 hpf (left, control; right, 0.5 mM pancuronium). An inset represents the head-tail angle (*θ*). Max turn angle and max turn speed of spontaneous coiling at 24 hpf are displayed. (Student’s *t*-test). **(C)** Superimposed images of touch response at 72 hpf (left, control; right, 0.5 mM pancuronium). An inset represents the head-turn angle (*θ*). Traveled distance, max turn angle and max turn speed of touch response at 72 hpf are displayed. (**p < 0.01, paired *t*-test).

Taking advantage of the dependence on distinct populations of muscles for locomotion at 24 and 72 hps, we examined the effect of pancuronium on slow and fast muscles. Coiling at 24 hpf and burst swimming at 72 hpf occuring spontaneously or elicited upon touch were recorded under stereomicroscope (Figure 1). The percentage of embryos judged to perform coiling or burst swimming was plotted against the concentration of pancuronium (Figure 1A). Burst swimming at 72 hpf was more sensitive, with only ∼20% of embryos responding to touch at 1 mM pancuronium. More than 80% of embryos at 24 hpf responded to the same concentration of pancuronium.

We also examined specific parameters of spontaneous coiling and touch response using a high-speed camera. All embryos at 24 hpf responded to touch at 0.5 mM pancuronium (Figure 1A). At 0.5 mM pancuronium, the maximum turn angle and the maximum turn speed of embryos in spontaneous coiling was not changed compared to 0 mM control (Figure 1B). In contrast, treatment in pancuronium significantly reduced distance traveled, max turn angle and max turn speed at 72 hpf (Figure 1C). These results suggest that slow muscles are more resistant to pancuronium compared to fast muscles.

### Synaptic currents at the neuromuscular junction

The different effects of pancuronium on locomotion at 24 and 72 hpf may result from its potencies for AChRs in slow and fast muscles, as we expected. Alternatively, the difference may arise from other factors such as the absorption and the metabolism of pancuronium. To directly test the effect of pancuronium on NMJs, we recorded end-plate currents (EPCs) from fast and slow muscle of zebrafish.

**FIGURE 2:**
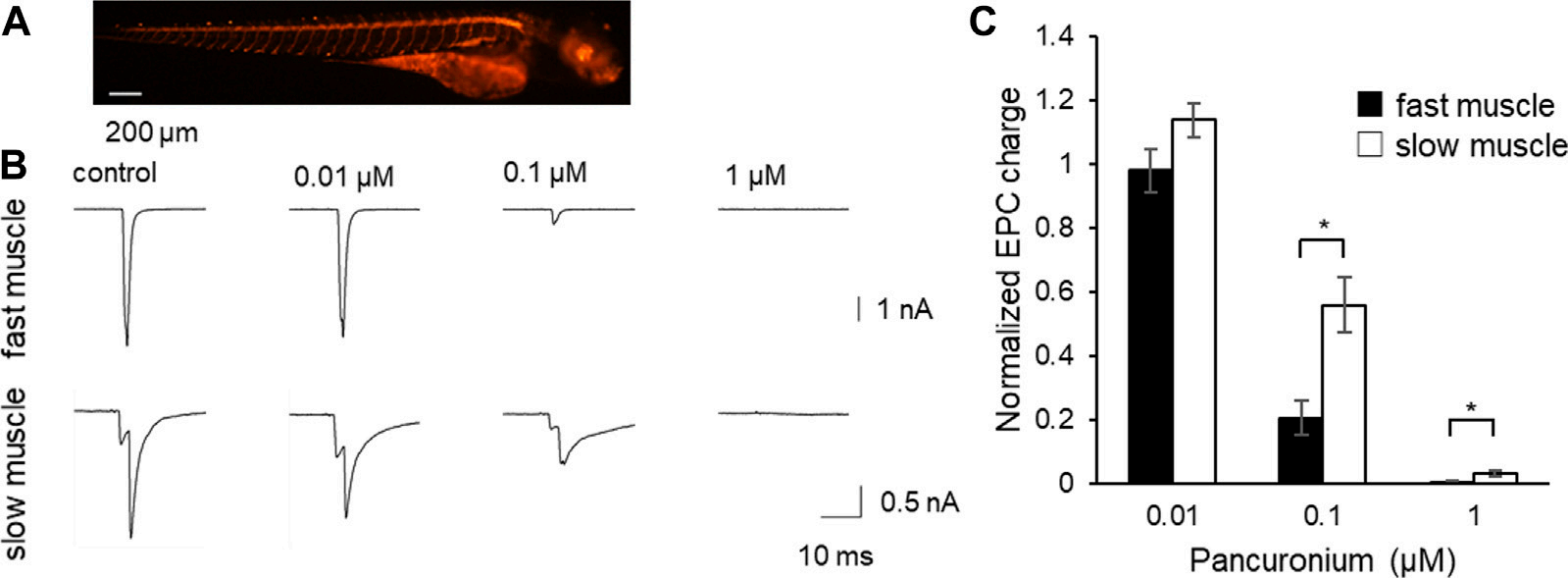
The effect of pancuronium on end-plate currents in fast and slow muscle fibers. **(A)** Fluorescent image of 4 dpf Tg (hb9:tTAad, TRE:ChRFR-TagRFP) zebrafish. Motor neurons specifically expressed channelrhodopsin fused with TagRFP. **(B)** Representative current traces from fast (top) and slow (bottom) muscle fibers in response to the light stimulation in the presence of pancuronium, whose concentration in the solution is indicated above the traces. Note some of the traces have notches, which result from the non-overlapping time course of inputs from multiple motor neurons innervating the muscle cell. (*p < 0.05, Student’s *t*-test with bonferroni correction) **(C)** The effect of pancuronium on EPC charges. Light-evoked EPCs were recorded in the varying concentration of pancuronium.

To perform the experiment, we expressed channelrhodopsin in motor neurons using a motor neuron specific promoter hb9. Channelrhodopsin was fused with TagRFP as an expression marker. A stable line harboring the clone was established, in which motor neurons expressed TagRFP signals (Figure 2A) at 4 dpf. Whole-cell patch-clamp recordings were performed from innervated fast or slow muscles. Blue light stimulation of motor neurons immediately depolarized all motor neurons, which induced them to generate action potentials. The EPCs recorded from voltage clamped muscle cells increased stepwise with the length of the light stimulus (data not shown), consistent with multiple innervation on a single muscle fiber in zebrafish (Westerfield et al., 1986). A robust current was triggered faithfully by 1 ms blue light stimulation both in fast and slow muscles.

Both at 0.1 and 1 μM pancuronium, the synaptic current was suppressed stronger in fast fibers (Figures 2B,C). This suggests that pancuronium has stronger potency for AChRs in fast fibers.

### Heterologous expression of zebrafish subunit clones

**FIGURE 3:**
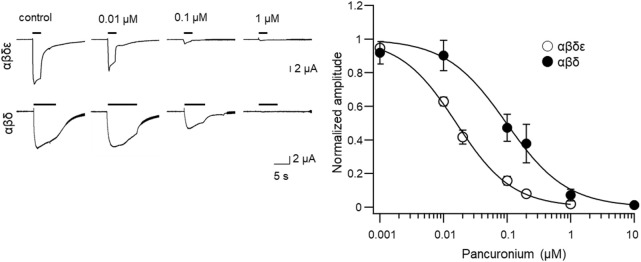
Concentration-dependent effects of pancuronium for inhibition of acetylcholine-induced current in oocytes expressing zebrafish fast (αβδε) or slow muscle-type (αβδ) nicotinic acetylcholine receptors.

Previous studies showed that AChRs in fast muscles of zebrafish are composed of αβδε or αβδγ, while those in slow muscles lack ε/γ (Mongeon et al., 2011). To test whether the different response to pancuronium observed in synaptic currents (Figure 2) was caused by different subunit compositions, we heterologously expressed two combinations of subunits, αβδε or αβδ, and examined the effects of pancuronium on the observed ACh-induced current. mRNA for each subunit was synthesized from zebrafish genes and injected into unfertilized Xenopus oocytes. After incubation for 2–3 days, AChR currents were measured under the two-electrode voltage clamp. Normalized ACh-induced current amplitude was plotted against the concentration of pancuronium. The fitted curve generated from the αβδε subunits was left-shifted compared to the αβδ subunits (Figure 3), reaching ∼0 at 1 μM (IC_50_ was 15.6 ± 1.1 nM for αβδε and 96.2 ± 20.3 nM for αβδ, *p* = 0.016, Student’s *t*-test). This result is in line with those of locomotion and synapse physiology (Figures 1, 2), and suggests that AChRs consisting of αβδε subunits in fast muscles are more sensitive to pancuronium than those in slow muscles comprising αβδ subunits.

## Discussion

In this study, we revisited a question which has remained unanswered since the 1960s, and showed that pancuronium is more effective on fast muscle fibers than on slow muscle fibers, due to the different subunit compositions of nAChRs associated with each fiber type.

The IC_50_ of pancuronium for slow fibers and fast fibers was comparable between synaptic currents (Figure 2) and heterologous expression in Xenopus oocytes (Figure 3). Compared to these preparations, the concentration of pancuronium necessary to inhibit locomotion was ∼10,000 times higher (Table1). The absorption and the metabolic dynamics of pancuronium in intact larvae may make the higher concentration in the surrounding solution obligatory to achieve the effective concentration in the NMJ. This difference is indeed comparable to the observation for glycine receptors when inhibited by its antagonist strychnine (David-Watine et al., 1999; Roy et al., 2012).

**TABLE 1:** IC_50_ for two types of AChRs estimated from three experiments.

	Locomotion (Figure 1)	Muscle (Figure 2)	Oocyte (Figure 3)
IC_50_ in fast muscle (αβδε)	0.61 mM	55.3 ± 10.6 nM	15.6 ± 1.1 nM
IC_50_ in slow muscle (αβδ)	3.36 mM	108.6 ± 14.0 nM	96.2 ± 20.3 nM

The difference between fast muscle (αβδε) and slow muscle (αβδ), on the other hand, was more pronounced in oocytes compared to muscles (Table 1). This difference may result from the environment in which receptors were expressed: e.g., post-translational modification or membrane properties. Accessibility of applied pancuronium to fast and slow muscles may also have contributed to the difference, because slow muscles are located near the surface whereas fast cells are located deeper (Luna et al., 2015; Zempo et al., 2020).

The α subunits, characterized by the presence of two vicinal cysteines, contribute to the ligand binding site for acetylcholine (Weber and Changeux, 1974; Noda et al., 1982; Kao et al., 1984; Neumann et al., 1984, 1986; Sine et al., 1990). Heterologous expression of murine or *Torpedo* clones revealed that the acetylcholine binding sites are located at the interfaces of the α/γ and the α/δ subunits (Kurosaki et al., 1987; Sine et al., 1990; Sine and Claudio, 1991; Sine, 1993). Pancuronium, a non-depolarizing relaxant, is generally supposed to compete with ACh for a common binding site. Our study showed the difference in the affinity between αβδε and αβδ (Figures 2, 3). The contribution of the *γ* subunit will be negligible at the developmental stages where behavior and synaptic physiology were analyzed (Figures 1, 2), based on the expression time course of the *γ* subunit gene (Naganawa and Hirata, 2011; Walogorsky et al., 2012). The main difference of the binding sites may therefore result from the α/ε interface, which does not exist in the αβδ composition. Alternatively, the binding of pancuronium may occur not at the ACh binding site but at an unexpected moiety of the AChR pentamer.

Zebrafish is a good system to examine the fiber-specific effects of muscle relaxants, thanks to its amenability to multiple techniques used in this study, as well as their segregated distribution of slow and fast fibers. It is also possible that the current observation for pancuronium may be extended to other types of muscle relaxants. It remains unknown whether mammalian slow fibers, or a subpopulation of them, have αβδ compositions similar to zebrafish NMJs. It will be interesting to examine whether NMJs in species other than zebrafish also contain AChRs with the αβδ composition, and have distinct potencies of muscle relaxants depending on the fiber type.

## Data Availability

The raw data supporting the conclusion of this article will be made available by the authors, without undue reservation.
